# Supplementary material for: FastCAR: fast correction for ambient RNA to facilitate differential gene expression analysis in single-cell RNA-sequencing datasets
Source: BMC Genomics. 2023 Nov 29;24:722. doi: 10.1186/s12864-023-09822-3 (PMC10687889; doi:10.1186/s12864-023-09822-3)
Supplement: Supplementary file 2 — Supplementary Material 2: Supplementary Figure 1: FastCAR correction does not strongly affect clustering. A) Effect of FastCAR on the UMAP and clustering in the same cells from bronchial biopsies. B) Jaccard index of the overlap of the cell contained in the clusters between corrected and non-corrected bronchial biopsies. The same cells cluster together even if the clusters get split differently along a gradient. Supplementary Figure 2: Comparison of applying different ambient RNA correction methods between asthma and control in selected cell types in bronchial biopsies. A) IGKC levels in selected expressing and non-expressing cell types without correction and after applying other correction methods. B) SCGB3A1 levels in selected expressing and non-expressing cell types without correction and after applying other correction methods [file 12864_2023_9822_MOESM2_ESM.pdf]

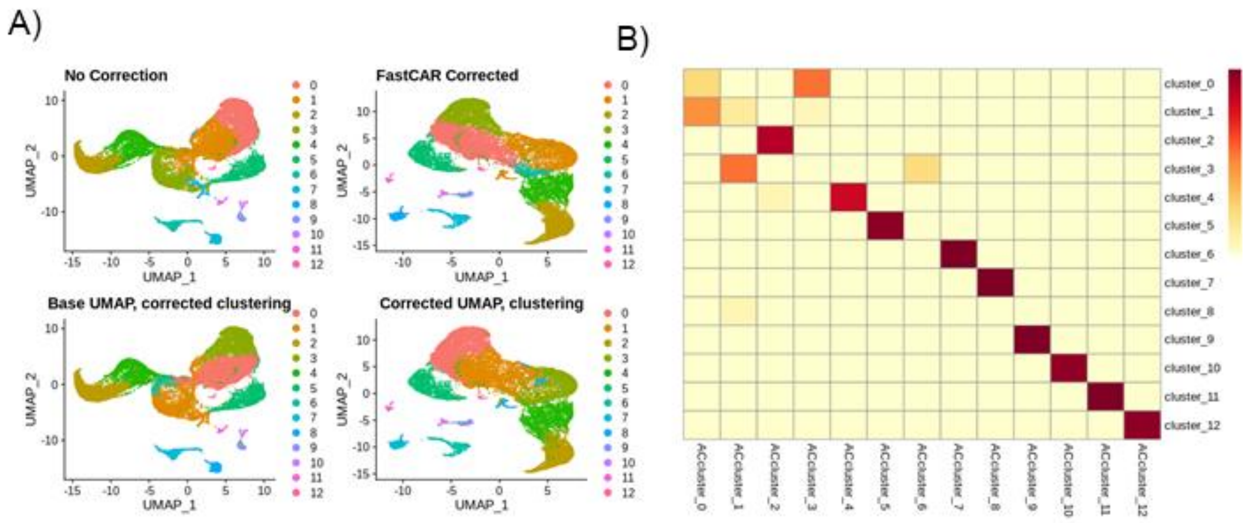

Supplementary Figure 1: FastCAR correction does not strongly affect clustering. ***A)*** *Effect of FastCAR on the UMAP and clustering in the same cells from bronchial biopsies.* ***B)*** *Jaccard index of the overlap of the cell contained in the clusters between corrected and non-corrected bronchial biopsies. The same cells cluster together even if the clusters get split differently along a gradient.*

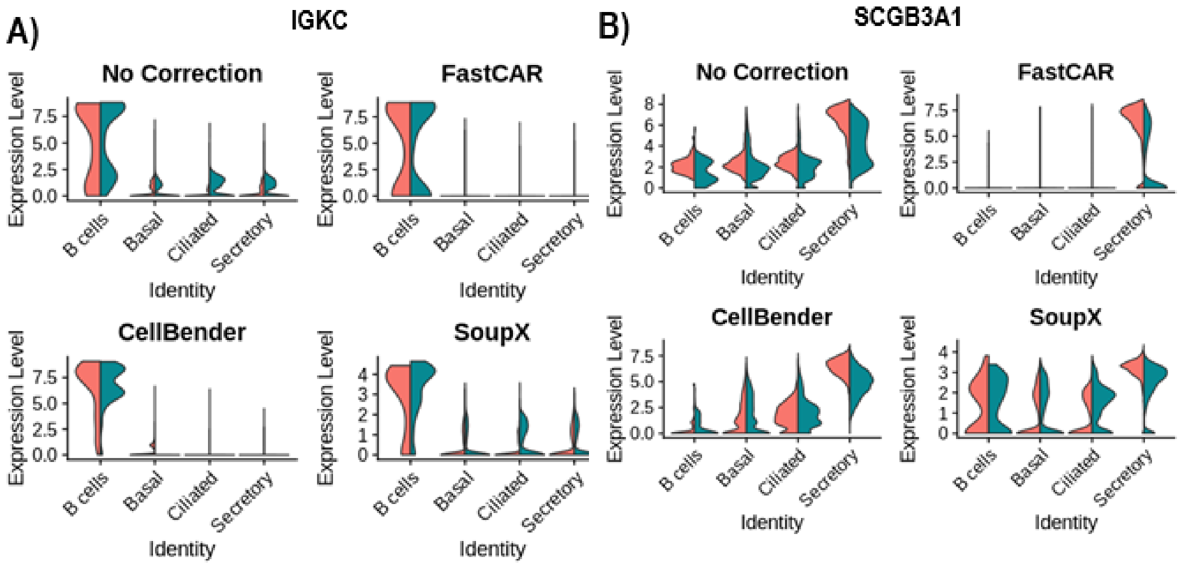

Supplementary Figure 2: Comparison of applying different ambient RNA correction methods between asthma and control in selected cell types in bronchial biopsies. ***A)*** *IGKC levels in selected expressing and non-expressing cell types without correction and after applying other correction methods.* ***B)*** *SCGB3A1 levels in selected expressing and non-expressing cell types without correction and after applying other correction methods.*
